# Fibroblasts alter the physical properties of dermal ECM-derived hydrogels to create a pro-angiogenic microenvironment

**DOI:** 10.1016/j.mtbio.2023.100842

**Published:** 2023-10-24

**Authors:** Meng Zhang, Fenghua Zhao, Xue Zhang, Linda A. Brouwer, Janette K. Burgess, Martin C. Harmsen

**Affiliations:** a University of Groningen, University Medical Centre Groningen, Department of Pathology and Medical Biology, Hanzeplein 1 (EA11), 9713, GZ Groningen, the Netherlands; b University of Groningen, University Medical Centre Groningen, W.J. Kolff Institute for Biomedical Engineering and Materials Science-FB41, A. Deusinglaan 1, 9713, AV Groningen, the Netherlands; c University of Groningen, University Medical Centre Groningen, Department of Biomedical Engineering-FB40, A. Deusinglaan 1, 9713, AV Groningen, the Netherlands; d University of Groningen, University Medical Centre Groningen, Groningen Research Institute for Asthma and COPD (GRIAC), Hanzeplein 1 (EA11), 9713, AV Groningen, the Netherlands

**Keywords:** Vascularization, Extracellular matrix, ECM hydrogel, Endothelial cells, Fibronectin, Collagen, Biomechanics

## Abstract

This study aimed to investigate the impact of fibroblasts (MRC-5) on the extracellular matrix (ECM) microenvironment of endothelial cells (ECs) during the vascularization of skin-derived ECM hydrogel in vitro. Two types of ECs were studied: human dermal microvascular endothelial cells (HMEC) and human pulmonary microvascular endothelial cells (HPMEC). Results showed that the presence of MRC-5 fibroblasts increased the stiffness of the hydrogel and led to larger fiber diameters and increased porosity. Extensive collagen fiber remodeling occurred in the ECM hydrogel with MRC-5 fibroblasts. Additionally, higher levels of fibulin-1 and fibronectin were deposited in the hydrogel when co-cultured with MRC-5 fibroblasts. These findings suggest that MRC-5 fibroblasts play a role in modifying the ECM microenvironment, promoting vascularization through dynamic ECM remodeling.

## Graphical abstract

**Figure:**
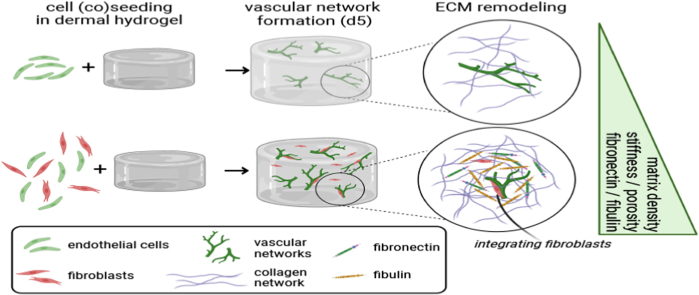

## 1 Introduction

The skin, the largest organ of the human body, is constantly exposed to external injuries. As a result, the importance of wound healing therapy becomes particularly relevant, especially in the case of patients with persistent chronic dermal wounds, such as those suffering from diabetes [1,2]. The process of dermal wound healing is intricate and involves four overlapping phases: hemostasis, inflammation, proliferation, and tissue remodeling or resolution [3,4]. The inflammatory and subsequent phases rely on adequate neo-vascularization (referred to as vascularization hereafter), which is the formation of new blood vessels [5,6]. These blood vessels facilitate the delivery of oxygen and nutrients and the disposal of waste products while also providing the gateway for the trafficking of regulatory immune cells [7].

The extracellular matrix (ECM) supports vascularization both biochemically and structurally [8,9]. The ECM acts as a reservoir for angiogenic factors, such as vascular endothelial growth factor (VEGF), angiopoietin 2, and fibroblast growth factors (FGF) [10]. These factors are bound to glycosaminoglycans (GAGs) in the ECM and are released in response to specific signals to stimulate the proliferation, migration [11,12], and differentiation of endothelial cells (ECs) [13]. Moreover, through adhesive interactions with integrins on the surface of ECs [14], ECM provides a scaffold that supports the growth and organization of ECs [10]. In addition to serving as a scaffold and supporting interaction with other cells, mechanical features of the ECM, such as stiffness, are sensed by virtually all cell types. They (mechano) transduce externally exerted forces to downstream pathways that regulate cell phenotype and function [15].

Mesenchymal cells like fibroblasts are activated during the reparative phase of wound healing and are pivotal in angiogenesis [16,17]. They secrete angiogenic growth factors, such as VEGF and FGF, which promote vascularization [18,19]. Previously, we showed that mesenchymal stromal cells from adipose tissue (ASC) co-cultured with large vessel endothelial cells, (Human umbilical vein endothelial cells，HUVECs) act as pro-angiogenic pericytes and promote vascular network formation (VNF) through paracrine and juxtracrine signaling both on culture plates [20] and in the three dimensional (3D) environment of Matrigel® [21]. In vitro, ASC not only deposit ECM components [22] but also degrade ECM through action of their secreted enzymes, including matrix metalloproteinases (MMPs) [23]. In skin, the main mesenchymal cell type is the fibroblast which is a ‘professional’ matrix remodeler. Fibroblasts newly deposit ECM components, including collagen I, collagen III, proteoglycans, fibulins and fibronectin [24,25]. The latter two are instrumental in vascularization processes. During ECM remodeling, fibroblasts also degrade ECM through secretion of MMPs [25], creating spaces and pathways for new blood vessels to grow and develop. This remodeling of ECM by fibroblasts regulates proliferation of ECs and stabilizes vessels. Matrigel® offers a 3D environment yet is basement-membrane-derived. Pure collagen type I hydrogels replicate the major ECM component of organs. Yet in our hands, collagen I hydrogels did not support vascularization by ECs irrespective of the presence of mesenchymal cells (unpublished data). Interestingly, human pulmonary microvascular endothelial cells (HPMEC) show vascularization in cardiac left ventricle ECM hydrogels irrespective of the presence of mesenchymal support cells [26], which raises the question what the role of mesenchymal cells is in vascularization by ECs in organ-derived ECM hydrogels. We hypothesized that mesenchymal cells remodel the ECM, altering the mechanical properties which augments vascularization.

In the present study, we examined the impact of matrix remodeling by MRC-5 fibroblasts on VNF by skin-derived microvascular endothelial cells (HMEC) and HPMEC (lung) in a skin-derived ECM hydrogel.

## 2 Materials and methods

### 2.1 Hydrogel synthesis

Porcine skin was purchased from a slaughterhouse (Kroon Vlees, Groningen, The Netherlands). The skin was cut into small pieces (1 cm^3^) and mixed with ice-cold Dulbecco's phosphate-buffered saline (DPBS) (Lonza Walkersville, Inc., Walkersville, MD, USA). The mixture was minced in a kitchen blender (Bourgini, Breda, The Netherlands) with DPBS until it formed a homogeneous paste. The tissue homogenate was sonicated using an Ultrasonic homogenizer (Sigma Aldrich, Amsterdam, the Netherlands) at 100 % power for 1 min, collected by centrifugation, washed using DPBS twice, and incubated with 0.05 % trypsin in DPBS (Thermo Fisher Scientific, Waltham, MA, USA) at 37 °C with constant shaking for 4 h (h). After washing twice with PBS, the slurry was incubated in Milli-Q® water with constant shaking at 37 °C overnight. Next, the tissue homogenate was treated with excess saturated NaCl (6 M) for 3 h. Subsequently, the homogenate was incubated in 1 % SDS (Sigma-Aldrich, St. Louis, MO, USA) and 1 % Triton X-100 (Sigma-Aldrich), followed by 1 % sodium deoxycholate (Sigma-Aldrich) and 30 μg/mL DNase (Roche Diagnostics GmbH, Mannheim, Germany) in 1.3 mM MgSO_4_ and 2 mM CaCl_2,_ Milli-Q® water was used as the base solution. All incubations were under shaking at 37 °C overnight. The crude ECM was washed three times with MilliQ® water between all incubations. Lastly, the homogenate was washed for an hour with DPBS under constant shaking, this process repeated six times, collected after centrifugation at 3000*g*, and 70 % ethanol was added for overnight sterilization at room temperature. The skin ECM samples were frozen in liquid nitrogen and lyophilized with a freeze dryer (Labconco, Kansas City, MO, USA) before being ground to a fine powder with an Ultra-Turrax homogenizer (IKA, Staufen, Germany). To generate hydrogels, 20 mg/mL of ECM powder was digested with 2 mg/mL of porcine pepsin (Sigma-Aldrich, St. Louis, MO, USA) in 0.01 M HCl with constant stirring at room temperature for 24 h. After digestion, the ECM was neutralized by adding 1/10th volume 0.1 M NaOH and subsequently 1/10th volume 10xDPBS to generate an isotonic and neutral pH ECM pregel which was stored at 4 °C until use.

### 2.2 3D cell culture

Both human microvascular endothelial cells (HMEC-1 [27], HMEC in the text) and human pulmonary microvascular endothelial cells (HPMEC-ST1.6R [28] HPMEC in the text, kind gift from Dr. Kirkpatrick, Johannes Gutenberg University, Mainz, Germany) were tagged with EGFP(green fluorescence) by third generation VSV-pseudotyped replication-deficient lentiviruses [29]. HPMEC were maintained in endothelial culture medium, consisting of RPMI-1640 (Lonza, Basel, Switzerland) with 20 % heat-inactivated fetal bovine serum (FBS, Sigma-Aldrich, MO, United States), 1 % penicillin/streptomycin (#15140122, Gibco Invitrogen, Carlsbad, CA, USA), 1 % l-glutamine (#17-605E, Lonza BioWhittaker, Verviers, Belgium), 5 U/mL heparin (LEO Laboratories Limited, Ballerup, Denmark), and 20 μg/mL of endothelial growth factors (EGF, home-made bovine brain extract [30]). HMEC were maintained in MCDB 131 (Gibco, Carlsbad, CA, USA) with 10 % FBS, 10 mM l-glutamine, 10 ng/mL EGF + 1 μg/mL hydrocortisone (Sigma, MO, United States). Primary lung fibroblast, MRC-5 [31], was lentiviral tagged with dTomato (red fluorescent) using third generation VSV-pseudotyped replication-deficient lentiviruses as describe above. The fibroblasts were maintained in Ham's F–12K (#BE12-615F, Lonza BioWhittaker, Verviers, Belgium) with 10 % fetal bovine serum and 1 % penicillin/streptomycin. For co-culture experiments, 0.5 × 10^6^ ECs and 0.5 × 10^6^ MRC-5 cells were suspended in 20 μL of RPMI-1640 (Lonza, Basel, Switzerland) which was carefully and homogeneously mixed with 200 μL of skin ECM pregel taking care to avoid trapping air bubbles. The resulting cell-gel mixtures were cast into individual wells of 48-well plates and incubated at 37 °C for 1 h to solidify the gel. Subsequently, 500 μL of endothelial culture medium was added to each well. As controls, hydrogels without cells were used, or hydrogels seeded with 0.5 × 10^6^ of each individual cell type.

### 2.3 Fluorescence cell imaging

After 5 days of culturing at 37 °C with 5 % CO_2_, inverted fluorescence microscopy (EVOS model M5000, Thermo Fisher) was used to acquire fluorescence micrographs to visualize the cells. ECs were visualized using GPF ‘light cubes’ (λ^ex^ 470/522 nm/λ^em^ 525/550 nm), and MRC-5 using Texas Red ‘light cubes’ (λ^ex^ 585/629 nm/λ^em^ 628/632 nm). VNF was further analyzed using densitometry, through processing the fluorescence micrographs with the Endothelial Tube Formation Assay—angiogenesis analyzer in Fiji [32]. It should be noted that the depth of field with the inverted microscope was restricted and only enabled assessment of the lower part of the hydrogels. The captured digital micrographs compressed the originally 3D situation onto a 2D plane which comprised the ability to distinguish between genuine branched tubes and tubes that crossed each other at different planes in the gel. While this caused a systemic error, and because no suitable 3D imaging and quantification software exists to date, we decided to process all images this way.

Live 3D scans of cell-seeded hydrogels were captured using a confocal laser scanning microscope Zeiss Cell Discoverer 7 imaging system (Zeiss, Jena, Germany). Optical settings were an objective lens magnification of 5 × and an Optovar magnification of 1 × . The interval of the scanning was 2.5 μm. Detection wavelengths (excitation–emission) were 450–550 nm for GFP and 550–700 nm for dTomato. Image stacks and videos were generated using Zeiss Zen 3.3 software.

### 2.4 Characterization of the mechanical properties

The gels loaded with cells were subjected to uniaxial compression with a 2.5 mm plunger at three locations [33,34], at least 2 mm away from the edge of the gel and ensuring 2 mm or more between each compression site. The stress relaxation test was performed with a low-load compression tester (LLCT) in ‘wet’ mode and at room temperature. The LLCT load cell and linear positioning for control and data were acquired using LabVIEW 7.1 software [35]. During compression, the increase in stress was continuously measured and the slope between the stress and strain curve was taken as the elastic modulus. Once the strain reached 0.2, it was maintained at this level for 100 s and the stress was continuously monitored. Percentage stress relaxation was calculated by comparing the stress at t = 0 s and t = 100 s.

### 2.5 Second harmonic generation (SHG) microscopy

Hydrogels were fixed with 2 % formalin for 24 h at 4 °C. Samples were embedded in 2 % agarose (Roche diagnostics, Indianapolis, USA) in PBS to prevent the hydrogel shrinkage during the dehydration process and dehydrated in a graded series of 50 %, 70 %, 80 %, 90 %, and 100 % ethanol, and incubated in xylol. The samples were paraffin-embedded and thin sections cut (4 μm) and mounted on glass slides. Subsequently, the slides underwent deparaffinized with xylene and a graded ethanol series to rehydrate. Microscopy Aquatex (Merck KGaA, Darmstadt, Germany) was used as mounting medium. Second harmonics generation (SHG) imaging was performed using a multiphoton laser confocal scanning microscope (model Zeiss 780, Zeiss, Jena, Germany). Generally, non-centrosymmetric molecules such as collagen will cause harmonics at half (425 nm) the wavelength of the incident light (850 nm) from which both forward and backward signals can be acquired. For this study backward SHG signals were acquired, and image analysis was performed using Fiji with macro TWOMBLI [36].

### 2.6 Hydrogel ultrastructure

Hydrogel ultrastructure was investigated with scanning electron microscopy (SEM). Upon conclusion of culturing, hydrogels were fixed with 2.5 % glutaraldehyde (111-30-8, Sigma, Darmstadt, Germany) and 2 % paraformaldehyde in PBS at 4 °C for 24 h. Then, the hydrogel was prepared following the procedures outlined in previously published literature [37]. Hydrogels were washed three times with DPBS and once with Milli-Q® water to remove any remaining fixatives and salts. Samples were dehydrated in the same way and embedded in paraffin as described above for the SHG samples. Then, 50 μm thick sections were cut and mounted onto glass coverslips (size 18 × 18 mm). After drying, the sections were deparaffinized in xylene and rehydrated in a series of 100 %, 96 %, and 70 % ethanol. Dry slides were glued on top of 6 mm SEM pin stubs (Agar Scientific, Stansted, UK) and Carbon coated with Leica EM ACE600 sputter coater device (Leica Microsystems B.V., Amsterdam, Netherlands). Hydrogels were visualized at 5000 × , 10,000 × , and 25,000 × magnification, at 3 kV with Zeiss Supra 55 STEM (Carl Zeiss NTS GmbH). Image analysis was performed using Fiji with plugin DiameterJ [38].

### 2.7 Immunofluorescence staining

Thin paraffin sections (4 μm) were deparaffinized and rehydrated. For antigen retrieval, slides were incubated in 10 mM citric acid (pH 6) at 85 °C overnight. Slides were washed with demi water and PBS and subsequently blocked in 4 % BSA for 15 min at room temperature. Afterwards, the slides were incubated 1.5 h with a rabbit anti-human fibronectin antibody (ab6584, Abcam, 1:100) or mouse anti-human a fibulin-1 antibody (ab211536, Abcam, 1:100) at room temperature. After that, the slides were washed in PBS 3 times and incubated with a secondary antibody; for fibronectin: goat anti-rabbit HRP (P0448, Dako, 1:200) and for fibulin-1:rabbit anti mouse (F0313, Dako, 1:200) for 1 h. Opal 650 (Akoya Biosciences, 1:200) diluted in 0.1 M borate buffer with 0.003 % hydrogen peroxide (Merck, Darmstadt, Germany) was added and the slides incubated for 15 min. The slides were washed with demi water 3 times and incubated with DAPI (4′,6-diamidino-2-phenylindole, Merck 1:5000) for 10 min. Images were generated using a SP8 confocal microscope (Leica, Wetzlar, Germany). The acquired staining images were subject to analysis using Fiji. Three lines were drawn at predetermined positions (top, middle, and bottom) across each image (Fig. S1A). Importantly, the positioning of these lines in the field of view remained constant across all images analyzed. The corresponding plot profiles for the fluorescence signals along these three lines were extracted by Fiji built-in function: plot profile. Each group included three images. All plot profiles were calculated with total area under the curve (TAUC) and peak area under the curve (TPAUC).

### 2.8 Statistical analysis

All statistical analyzes were performed using GraphPad Prism v9.2.0 (GraphPad Company, San Diego, CA, USA). All data were scrutinized for outliers using the robust regression and outlier removal (ROUT) test. All data except LLCT data were analyzed with one-way ANOVA. LLCT data were analyzed with two-way ANOVA. Differences were considered significant at p < 0.05 in the corresponding statistical tests.

## 3 Results

### 3.1 Fibroblasts promote endothelial vascular network formation in skin-derived ECM hydrogels

**Fig. 1:**
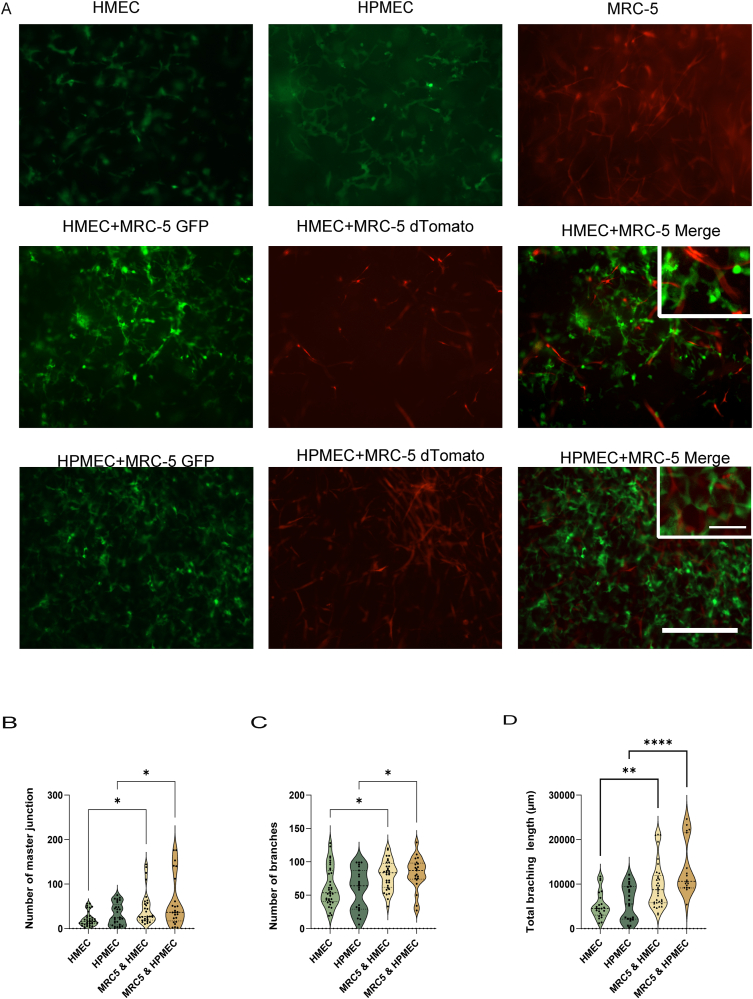
Vascular network formation (VNF) by endothelial cells (ECs) in a 3D culture system either alone or in co-culture with MRC-5 cells. (A) The EGFP-expressing human dermal microvascular endothelial cells (HMECs) or human pulmonary microvascular endothelial cells (HPMEC) (green) and dTomato-expressing MRC-5 fibroblasts (red) were cultured in 48-well plates for five days. Scale bar - 400 μm. (B) Comparison of the number of master junctions based on Fiji quantification of VNF by ECs either alone or in co-culture with MRC-5 in skin extracellular matrix (ECM) hydrogels. (C) Comparison of the number of branches of VNF by ECs either alone or in co-culture in skin ECM hydrogels. (D) Comparison of total branching length based on Fiji quantification of VNF by ECs either alone or in co-culture with MRC-5 in skin ECM hydrogels. The data are from 7 independent experiments, while 3 different randomized regions were measured for every sample, each dot represents a measurement of a randomized region. Statistical testing by one-way ANOVA, *p < 0.05,** p < 0.01,**** p < 0.0001. (For interpretation of the references to color in this figure legend, the reader is referred to the Web version of this article.)

Formation of branched and extensive VNF by HMEC and HPMEC had occurred in skin-derived ECM hydrogels after 5 days of culturing (Fig. 1A). The VNF (Fig. 1A, green) also occurred in the presence of fibroblasts (MRC5, Fig. 1A, red) where the fibroblasts often aligned closely to the endothelial branches (Suppl. Figs. S2A and B).

The extent of the VNF was quantified to determine the number of master junctions (cells linking at least three branches), the number of branches, and the total branching length (resp. Fig. 1B, C and D). The analysis of VNF formed by ECs were delineated as illustrated in Suppl. Fig. S2C. Fibroblasts increased the number of master junctions in HMEC networks compared to HMEC alone (resp 45.9 ± 38.2 vs 21.2 ± 15.9, p < 0.05), and increased the number of master junctions produced by compared to HPMEC alone (resp 58.9 ± 56.9 vs 31.7 ± 24.4, p < 0.05) (Fig. 1B). In addition, fibroblasts enhanced the number of branches generated by the ECs. Specifically, fibroblasts augmented branching by HMEC (80.2 ± 21.9), compared to HMEC alone (62.1 ± 29.3, p < 0.05). Similarly, fibroblast boosted branching by HPMEC, 82.9 ± 24.4 branches vs 57.7 ± 31.3 branches by HPMEC alone (p < 0.05, Fig. 1C). Fibroblasts augmented the total branching length within the VNF (Fig. 1D) by HMEC (9703 ± 5125 μm vs 5217 ± 3001 μm in controls, p < 0.01) and HPMEC (13551 ± 6102 vs 5670 ± 3965 μm in controls, p < 0.0001) compared to the ECs alone.

### 3.2 Fibroblasts acquire a pericytic position on endothelial branches in skin-ECM hydrogels

To further dissect the observed alignments and interactions of fibroblasts with endothelial tubes, real time 3D confocal laser scanning fluorescent microscopy (Zeiss Cell Discoverer 7) was used. The green channel was used to visualize the EGFP-tagged ECs, while the red channel was used to visualize the dTomato-tagged MRC-5 fibroblasts (Suppl. Video S1). The video shows that branches of the tubes extended in all three dimensions and throughout the hydrogel. The meshes created by ECs had expanded to form a reticulated vascular-like network, to which the fibroblasts had aligned. Additionally, the fibroblasts had stretched into the ECM, indicating that these were in the process of remodeling the ECM that surrounded ECs and their network.

### *3.3* The changes of physical properties of skin ECM hydrogels by VNF is affected by fibroblasts

**Fig. 2:**
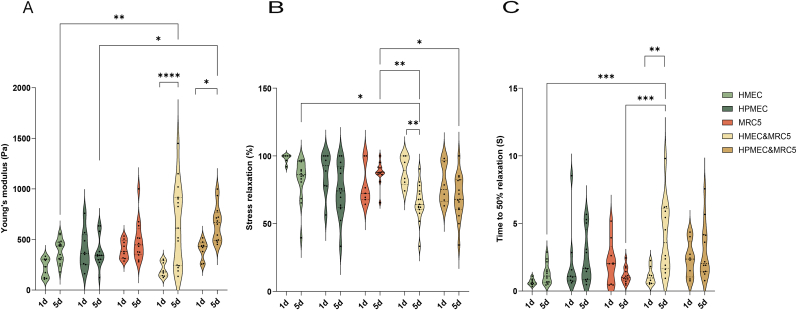
Comparison of physical characteristics of skin ECM hydrogel loaded with ECs either alone or in co-culture with MRC-5 cells after one- and five-days culture. (A) Stiffness of skin ECM hydrogel loaded with ECs either alone or in co-culture with MRC-5 cells on days one and five. Skin ECM hydrogels were tested using low-load compression tester (LLCT) with a fixed 20 % strain ratio. (B) Total stress relaxation of skin ECM hydrogel loaded with ECs either alone or in co-culture with MRC-5 cells on days one and five. After compressing the skin ECM hydrogel using LLCT with a fixed 20 % strain ratio, the stress relaxation was recorded in 100s. (C) Time to 50 % relaxation of skin ECM hydrogel loaded with ECs either alone or in co-culture with MRC-5 cells on day 1 and day 5. The data are from resp. three and five independent experiments for days one and five while three randomly selected spots on the hydrogel were measured for every single sample, and each dot represents a measurement. Statistical testing by two-way ANOVA, *p < 0.05, **p < 0.01,*** p < 0.001,**** p < 0.0001.

The stiffness of cell-loaded hydrogels was measured using a low-load compression tester (LLCT). After five days of culture, the stiffness of hydrogels containing ECs co-seeded with MRC-5 increased (Fig. 2A). In contrast, the stiffness of control gels i.e. seeded with only fibroblasts or ECs alone did not differ compared to day 1. Hydrogels containing co-cultures of fibroblasts and ECs had an almost twofold higher stiffness (resp. HMEC & MRC-5 and HPMEC & MRC-5: 651.7 ± 403.1 Pa and 661.9 ± 180.7 Pa) compared to controls (resp. HMEC and HPMEC: 386.9 ± 115.5 Pa, p = 0.0055, and 383.7 ± 155.3 Pa, p = 0.0111).

Besides the resistance to deformation i.e. stiffness (*E*), hydrogels comprise of a viscous component that dictates stress relaxation (σ). This is the time-dependent decrease in stress under a constant strain. Both stiffness and stress relaxation of hydrogels were determined by compression testing after 20 % strain and relaxation for 100s. After one day of culture, stress relaxation for all cell-loaded hydrogels was similar and reached close to 100 % stress relaxation (Fig. 2B). Prolonged culturing (5 days) resulted in decreased stress relaxation in HMEC & fibroblast-seeded hydrogels compared to day 1 (Fig. 2B, resp. 66.8 ± 10.8 vs 86.7 ± 10.7, p = 0.0024). At day 5, hydrogels with fibroblasts co-cultured with HMEC or HPMEC had the largest reduction in stress relaxation compared to MRC-5 group (Fig. 2B, resp. 66.8 ± 10.8 vs 87.3 ± 8.4, p = 0.0045 and 69.6 ± 16.3 vs 87.3 ± 8.4, p = 0.0347). Stress relaxation of hydrogels with fibroblasts co-cultured with HMEC decreased compared to that in hydrogels with HMEC on day 5 (66.8 ± 10.8 vs 82.8 ± 15.78, p = 0.0218).

Time to 50 % stress relaxation is a surrogate measure for hydrogel strain release dynamics. After one day of culture, time to 50 % relaxation did not differ between the different cell-seeded hydrogels yet the variation was large. The presence of fibroblasts and HMEC in the hydrogel increased time to 50 % relaxation on day 5 compared to day 1 (Fig. 2C, 4.1 ± 2.7 vs 1.1 ± 0.6, P = 0.0012). Hydrogels of fibroblasts co-seeded with HMEC tended to relax slower than HMEC only seeded hydrogels (4.1 ± 2.7 vs 1.3 ± 0.8, p < 0.001) after 5 days of coculture, also hydrogel containing fibroblasts with HMEC increased time to 50 % relaxation compared to MRC5 alone hydrogels (Fig. 2C, 4.1 ± 2.7 vs 1.2 ± 0.6, p < 0.001).

### 3.4 Remodeling of skin ECM hydrogels during VNF is augmented by fibroblasts

**Fig. 3:**
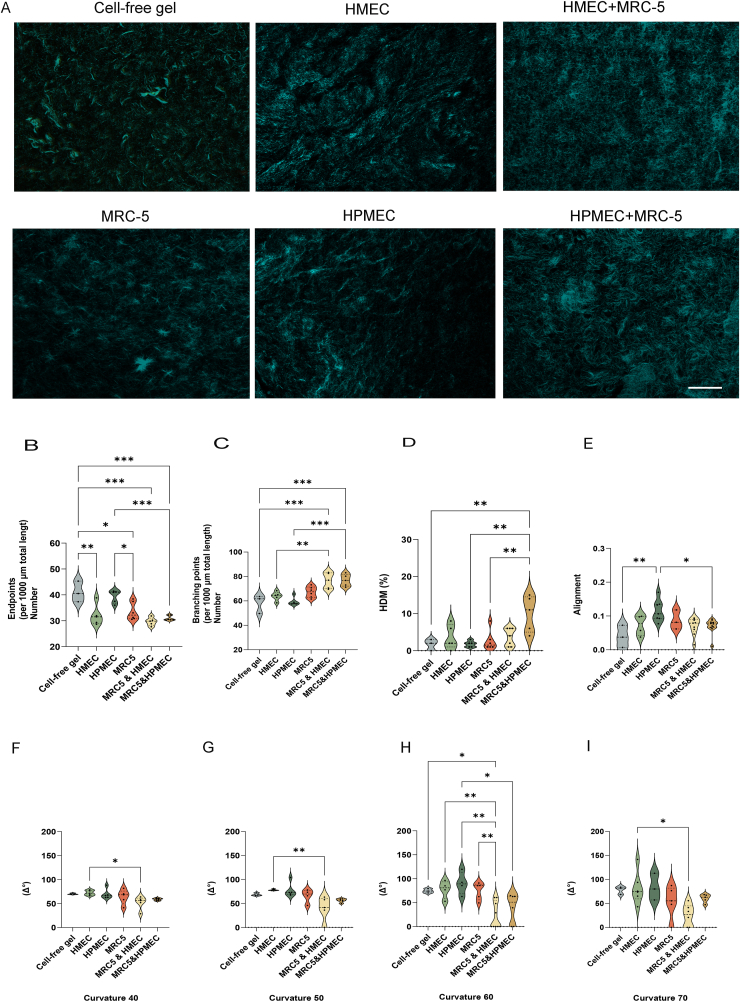
Remodeling of skin ECM hydrogels during VNF is augmented by fibroblasts. (A) Collagen fibers were detected by second harmonic generation (SHG) microscopy (scale bar - 50 μm). (B) Endpoints per 1000 μm total length. (C) Branching points per 1000 μm total length. (D) Percentage High-Density Matrix (HDM). (E) Fiber alignment. (F – I) Curvatures of resp. 40, 50, 60 and 70 arbitrary units. The data are generated from five independent experiments while each dot represents a measurement on one SHG micrograph. Statistical testing by one-way ANOVA, *p < 0.05, **p < 0.01.***p < 0.001.

Second harmonic generation by 2-photon scattering confocal microscopy is an established method to visualize changes of collagen fiber architecture [39,40]. Compared to blank skin-derived ECM hydrogels, the presence of cells altered the collagen architecture and increased the fiber density (Fig. 3A). In a bird's eye view the micrographs showed that after five days of culture fibroblast-seeded hydrogels did not differ from cell-free hydrogel controls (Fig. 3A). In contrast, five days of VNF led to a condensation of collagen fibers which appeared to be stimulated by fibroblasts (Fig. 3A), irrespective of the endothelial source. Hydrogels co-seeded with fibroblasts and ECs showed shorter and denser collagen fibers compared to the ECs only control hydrogels. In contrast, the ECs only hydrogels displayed longer and thicker bundles of collagen fibers, with distinct intersecting gaps compared to the co-seeded groups. The collagen architecture was assessed by densitometry (Fig. 3B–J) using the TWOMBLI plugin in FIJI which generates several surrogate parameters as presented in the following. In general, these analyses confirmed the gross observations of the micrographs and showed that collagen architecture (endpoints, branching points, high density matrix (HDM) and fiber alignment) of cell-free gel was altered in ECs seeded gel during VNF. While collagen rearrangement was augmented by fibroblasts during VNF (Fig. 3B–E). Fibroblasts increased the density of the fibers in the co-cultured groups. While the number of branching points increased when fibroblasts were together with HMEC or HPMEC compared to ECs controls (HMEC & MRC5 vs HMEC: 76.6 ± 6.5 vs 63.9 ± 3.634, p = 0.0063; HPMEC & MRC5 vs HPMEC: 76.85 ± 4.894 vs 59.50 ± 3.480 p =0.0002, Fig. 3C). Moreover, fibroblasts increased the percentage of areas of high density of fibers in HPEMC-seeded hydrogels compared to HPMEC alone (10.0 ± 4.8 vs 1.8 ± 0.8, p = 0.0021, Fig. 3D). Fibroblasts also changed the patterns of the fiber arrangement. Fibroblasts decreased the alignment of the fibers in HPEMC-seeded hydrogels compared to HPMEC gels (0.07 ± 0.02 vs 0.12 ± 0.03, p = 0.0185, Fig. 3E). The curvature of the collagen fibers, finally, is a parameter to reveal architectural remodeling and determines wave-like shapes. In other words, if fibers have a high frequency of bends and curves, this reflects as a high number below the arbitrary cutoff of 40 units. While above this threshold, this number is indicative of the wave amplitude, particularly for less ‘wavy’ fibers. Below 40 the curvature of the collagen fibers did not differ irrespective of the presence of cells. However, above the threshold, the curvature was higher in hydrogels loaded with HMEC alone compared to those co-cultured with fibroblasts and HMEC. This observation indicates that the fibers architecture was altered by cells (Fig. 3F–I).

### *3.5* Fibroblasts promote pericellular matrix porosity through fiber condensation

**Fig. 4:**
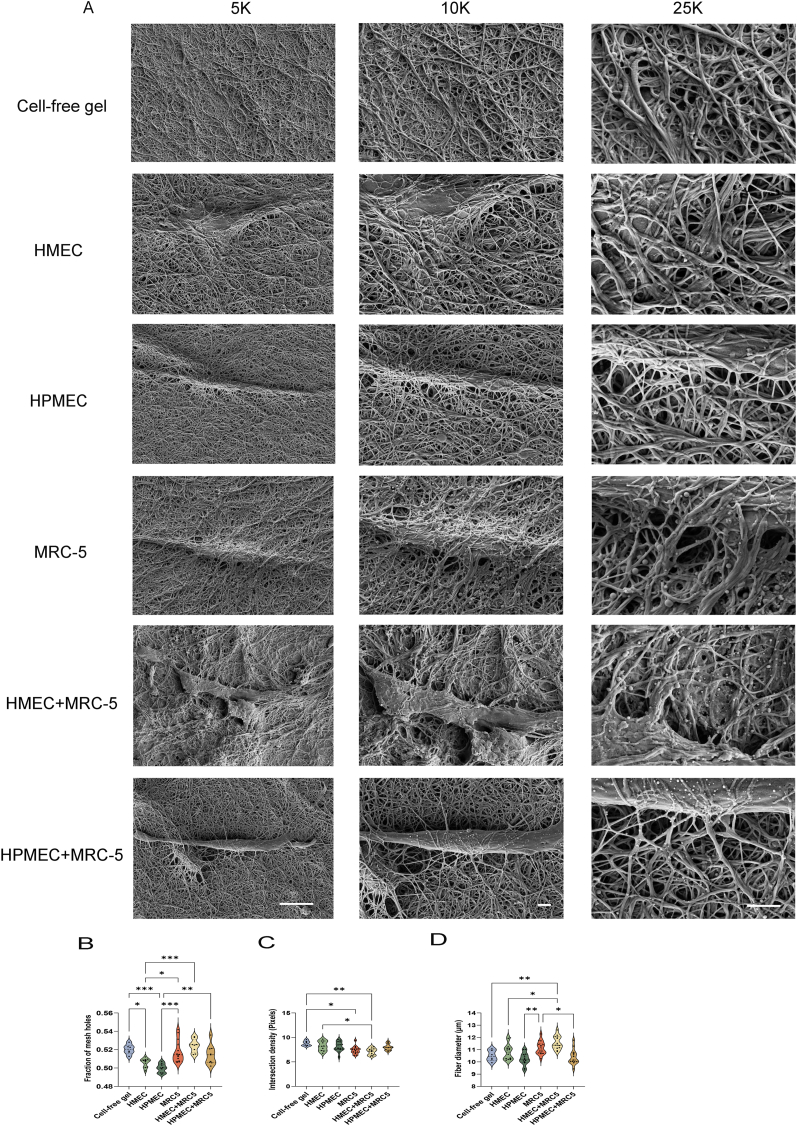
Ultrastructure of the extracellular matrix and analyses of the microstructure of the fibers and pores. (A) Fibers of the matrix were visualized by scanning electron microscopy (SEM) at three different magnifications: 5000 (5k), 10,000 (10k), and 25,000 (25k). Scale bars represent 10 μm in 5k micrographs and 2 μm in both 10k and 25k micrographs. (B) Mesh hole analysis of the fibers at 25k magnification. Percentage of porosity is the total number of holes pixels divided by the total image resolution. (C) Intersection density of the fibers at 25k magnification defined as [10,000 x (number of fiber overlaps)/(Total number of pixels in the micrograph)]. (D) Mean fiber diameter (μm). The data were generated from three independent experiments while three randomly selected regions were measured for every single sample, each dot represents a measurement of a randomized region. Statistical testing by one-way ANOVA comparing gel, *p < 0.05, **p < 0.01.***p < 0.001.

The ultrastructure of the hydrogel was visualized by SEM (Fig. 4A). Irrespective of the presence of cells, the skin-derived ECM hydrogels comprised a network of more or less randomly organized fibers that were visible already at low magnifications (Fig. 4A, left columns). At higher magnifications, the fibers had variable thickness (Fig. 4A, right columns). The fibers showed the typical striped repeat pattern typical for collagen fibers (Suppl. Fig. S3). The matrix in the immediate surroundings of fibroblasts or endothelial tubes was reorganized by apparent thickening of the fibers creating pores (Fig. 4A, right columns). Fibers surrounding the cells, were assessed in 25,000-fold (25k) micrographs. The ‘percentage of pores’ corresponds to the proportion of pore area relative to the entire image. ECs had reduced the fraction of mesh holes compared to hydrogels without cells (HMEC 0.52 ± 0.006 vs 0.51 ± 0.005, p = 0.0216, and HPMEC 0.52 ± 0.006 vs 0.50 ± 0.005, p = 0.0004. Fig. 4B). While the mesh holes around fibroblasts did not differ from control hydrogels without cells, their co-culture with ECs increased the fraction of mesh holes compared to cell-free or EC-seeded hydrogels. The intersection density (the number of fiber overlaps divided by the total number of pixels in the assessed area) was affected by fibroblasts. The intersection density of fibers within hydrogels co-seeded with fibroblasts and HMEC was lower compared to hydrogels containing HMEC alone. (Lower than hydrogel without cells, Fig. 4C). ECs or fibroblasts alone, had no influence on the average collagen fiber diameter compared to cell-free hydrogels (Fig. 4D). Yet, fibroblasts co-seeded with the different types of ECs had opposite influences: fibroblast-HMEC co-cultures had increased collagen fiber diameters compared to HMEC-seeded or cell-free hydrogels. In contrast, HPMEC-fibroblast co-cultures had reduced collagen fiber diameters compared to fibroblasts alone, but these were similar to those seen with HPMEC alone (Fig. 4D).

### 3.6 Fibroblasts promote deposition fibronectin and fibulin-1 during VNF

**Fig. 5:**
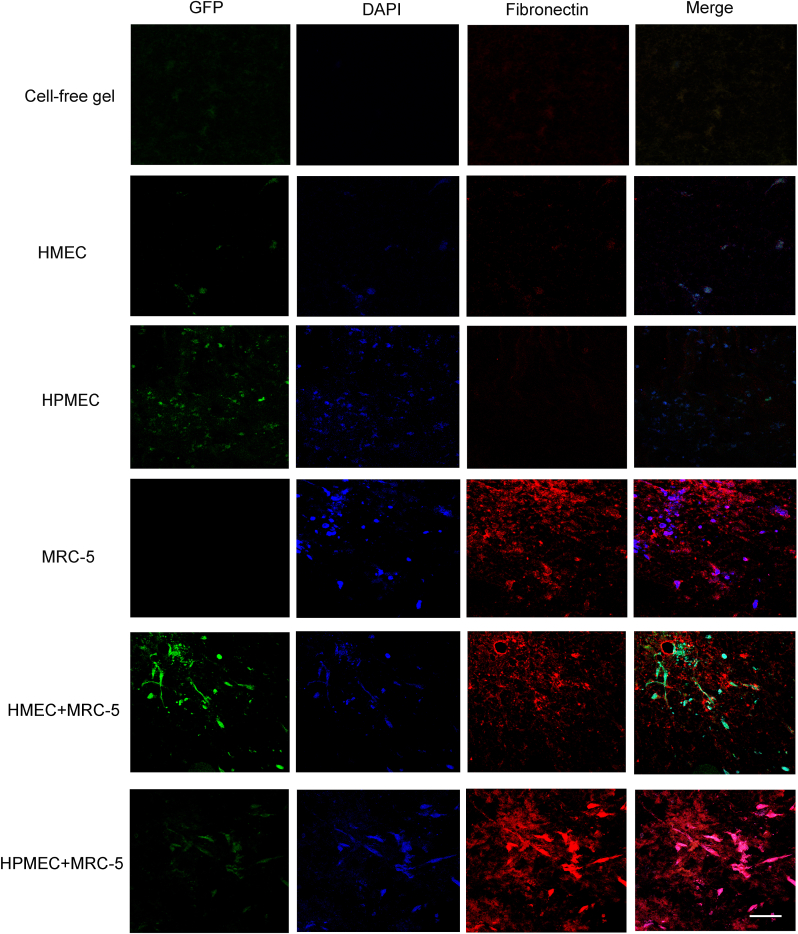
Fluoromicrographs of fibronectin-staining. Representative images of fibronectin staining of 4 μm sections of paraffin-embedded hydrogels. Merged images: green – GPF-labeled HMEC and HPMEC, red – fibronectin, blue – nuclei (DAPI). Scale bar: 58 μm. (For interpretation of the references to color in this figure legend, the reader is referred to the Web version of this article.)

Cell-free skin-derived ECM hydrogels contained no detectable fibronectin, and during VNF by HMEC or HPMEC fibronectin deposition was minimal (Fig. 5). In contrast, fibroblasts deposited large amounts of fibronectin after five days of culture in the hydrogel (Fig. 5). Fibroblasts appeared to promote fibronectin deposition during VNF by either EC type compared to during EC VNF alone, yet this did not differ from the amount of fibronectin deposition by fibroblasts alone (Fig. 5). The cross-sectional fluorescence intensity plot profiles (Suppl. Fig. S4A) showed that the peaks of the curves, representing a high density of fibronectin deposition, coincided with the presence of cells. The curves obtained from the histograms representing the co-cultures and fibroblasts seeded alone showed more peaks and higher peak values compared to those from the ECs-seeded hydrogels and the cell-free hydrogel groups (Suppl. Fig. S4A). To quantify and compare the influence of fibroblasts on the deposition of fibronectin, the area under the curve were determined (Suppl. Figs. S4B and C). The TAUC (this sums positive peaks, negative peaks, peaks that are not high enough to count, and peaks that are too narrow to count, Suppl. Fig. S4B) and the TPAUC (the area under every peak, Suppl. Fig. S4C) were calculated, and both areas were larger in the co-culturing groups than in the ECs cultured alone group, suggesting a greater deposition of fibronectin surrounding the ECs in the presence of fibroblasts, in contrast to the control groups consisting of a cell-free gel and an ECs-only gel.

**Fig. 6:**
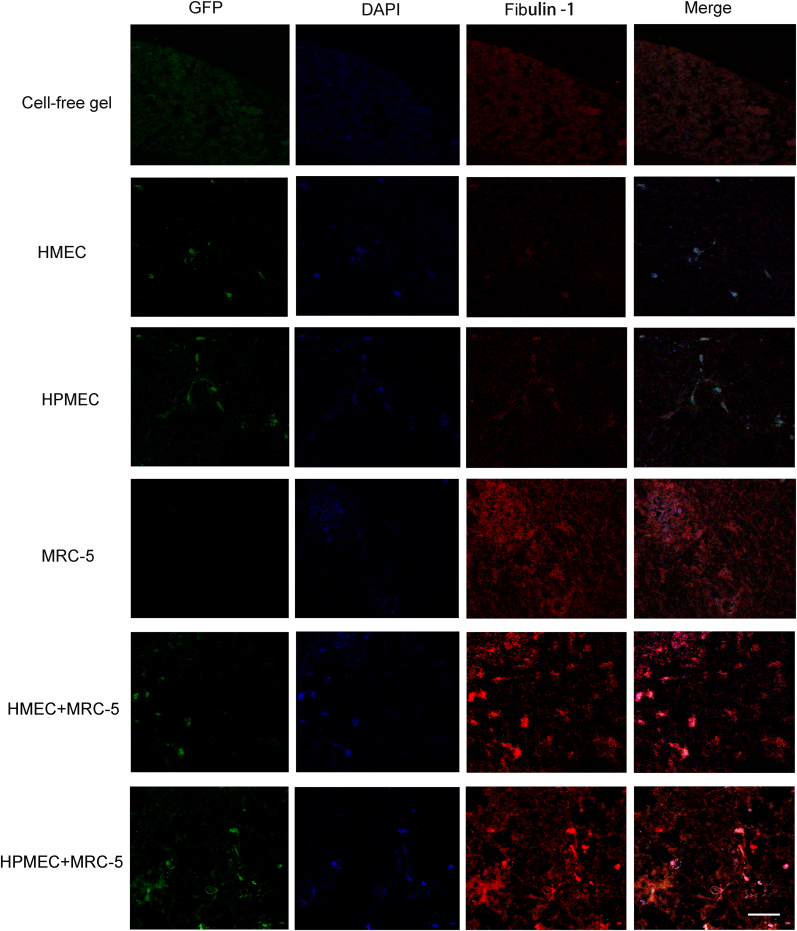
Fluoromicrographs of fibulin-1 staining. Representative images of fibulin-1 staining of 4 μm sections of paraffin-embedded hydrogels. Merged images: green – GPF-labeled HMEC and HPMEC, red – fibronectin, blue – nuclei (DAPI). Scale bar: 58 μm. (For interpretation of the references to color in this figure legend, the reader is referred to the Web version of this article.)

Besides fibronectin, fibulin-1 is also involved in vascularization processes. The immunostained fluoromicrographs (Fig. 6) showed that fibulin-1 had a deposition pattern similar to fibronectin (Fig. 5). Cell -free skin ECM hydrogels and EC-seeded hydrogels contained negligible amounts of fibulin-1, while ECs co-cultured with fibroblasts, deposited greater amounts of fibulin-1 (Fig. 6). The cross-sectional quantitative densitometry revealed that fibulin-1 was deposited solely around cells, irrespective of them being in single or co-culture (Suppl. Figs. S4D–F). The qualitative assessments of the fluorographs were corroborated by both the AUC for the intensities of fibulin-1, as well as their intensity peaks: irrespective of EC type, their co-culture with fibroblasts markedly increased fibulin-1 deposition (Suppl. Figs. S4E and F).

## 4 Discussion

In this study, we tested the hypothesis that fibroblasts promote vascularization and remodeling of skin-derived ECM hydrogels. Our main results were that the presence of fibroblasts alters the architecture and mechanical properties of ECM hydrogels, whilst also changing the biochemical ECM constituents. The altered architecture by fibroblasts resulted in changes of porosity of the ECM fiber meshes and diameter during VNF by microvascular ECs. Fibroblasts engaged in intimate contact with newly formed endothelial tubes akin to pericytes. Furthermore, the organization and alignment of collagen fibers within the skin ECM hydrogel were also altered by the interaction of fibroblasts with ECs. The biochemical composition of the ECM hydrogel was modified through the fibroblasts' deposition of fibronectin and fibulin-1.

In our study, we discovered that ECs have the ability to form vascular structures in skin-derived ECM hydrogels and that fibroblasts enhance VNF formation. We also observed that skin ECM hydrogel provides a conductive environment for VNF formation, which may serve as a model for in vivo vascularization e.g. during wound healing. This suggests that skin-derived ECM hydrogels are a suitable biomaterial that replicate several properties of skin ECM in vivo.

The impact of fibroblast-induced matrix remodeling and mechanical signaling on angiogenesis is less well-understood than the impact of e.g. hypoxia and growth factor signaling. An earlier study suggested that EC tube formation in 3D fibrin gels was primarily influenced by the presence of surrounding fibroblasts, rather than the composition of the surrounding medium [41]. In our study, as visualized in the 3D imaging, a proportion of the MRC-5 fibroblasts aligned closely with the ECs. Whisler et al. observed that there was a physical interaction between ECs and fibroblasts cocultured in fibrin hydrogel, as evidenced by the elongation of fibroblasts along the external walls of vascular tubes [42]. This implies that fibroblasts may affect VNF formation through juxtracrine signaling or other factors, such as modulation of the ECM, rather than solely through fibroblast-derived paracrine factors.

The formation of highly branched vascular networks, in pure collagen hydrogels, correlates with the mechanical properties of these hydrogels [43,44]. Previously, we showed that microvascular ECs (HPMEC) engage in VNF in cardiac-derived ECM hydrogels in a seeding density and time-dependent fashion [26]. Thus, VNF likely depends both on the physical and biochemical nature of the surrounding matrix. Indeed, we also showed that fibroblast-augmented VNF increased the stiffness of the skin-derived ECM hydrogel, while simultaneously reducing its stress relaxation. This suggests that part of the increased VNF is due to fibroblasts that actively remodel the ECM. In contrast, others showed that an increase in ECM density reduces the number of vascular tubes, yet it promotes the formation of thicker, deeper networks and more stable lumens in other biomaterials such as mixed collagen-fibronectin hydrogels which are much stiffer materials [45,46]. On the other hand, an increased ECM density hindered network formation and adding fibroblasts to EC-embedded 3D gels resulted in increased VNF regardless of matrix density [47]. As for the definition of higher stiffness, variations in the Young's modulus range were observed across these studies, attributable to disparities in the methodologies employed for stiffness assessments and the diverse materials under consideration [48]. This conflicting evidence might pertain to the challenge of quantifying 3D VNF. Moreover, we observed a greater variability in stiffness within hydrogels in which fibroblasts were co-cultured with ECs in comparison to groups with ECs cultured independently. A study by Juliar et al. demonstrated that the co-culture of fibroblasts with ECs induced stiffness heterogeneities in fibrin hydrogels during VNF [49]. The substantial variability in stiffness of skin ECM hydrogel may also be caused by the effects of fibroblast on the peri-endothelial stiffness. This effect may be influenced by the new generated ECM-binding proteins, such as fibronectin and fibulin. In addition, previous research examining co-culture of fibroblasts and ECs within synthetic polyethylene glycol (PEG) hydrogels also demonstrated a correlation between fibronectin deposition and the stiffening of the hydrogel [50].

Natural biomaterials possess viscoelastic properties, characterized by the presence of both an elastic component (stiffness) and a viscous component, which are both key to vascularization [51]. However, the impact of matrix viscosity on ECs is relatively unexplored. In general, the stress relaxation rate of ECM-derived biomaterials decreases with increasing stiffness. In our study, no differences in viscosity were observed between any of our hydrogels after day 1. However, after 5 days of culturing, the viscosity of the hydrogels containing fibroblasts co-cultured with ECs was higher compared to the groups with ECs cultured alone. We suggest that this increase in viscosity may be attributed to the degradation/rearrangement of ECM proteins during the VNF process, which potentially increased the fluidity of the ECM hydrogel.

In this study, we employed second harmonics generation microscopy to investigate architectural changes in collagen fibers at the macroscale level, which has not been previously addressed in other published studies related to 3D culturing in biomaterials. Collagens, particularly types I and III, are the most abundant ECM proteins in the skin, and both exhibit SHG properties. SHG imaging is a valuable technique to examine ECM structure [40,52,53]. High-resolution images from backward propagating SHG can be used to identify newly synthesized immature collagen or collagen turnover in older tissues. In our study, the collagen fibrils observed in the cocultured groups exhibited high density. This coincided with the abundant deposition of newly formed, fibronectin and fibulin-1 by fibroblasts which likely contributed to the observed increased matrix density. The ultrastructural analyses also showed that fibroblasts appear to facilitate migration of ECs and tube formation through degradation of the ECM causing an increased porosity around VNF areas. In contrast, in the hydrogels that only contained ECs, collagen fibrils exhibited a relatively high degree of alignment, forming entangled bundles composed of long fibers. These newly formed fibrils displayed a heterogeneous distribution, filling the pores between the fibers observed in SEM images and impeding the migration and interconnection of ECs.

The nanometer to micrometer-scaled topography of ECM-based hydrogels is dictated by the arrangement and structural attributes of fibers. Altogether, these characteristics dictate the architecture, geometry, pores and interconnectivity which are all sensed by embedded cells. The topography influences cellular phenotype and function such as ECM remodeling by fibroblasts and vascularization by ECs [54,55]. VNF might be enhanced by physical nano topography, while also being dependent on the cell type. Our research showed that the achieved diameter of the skin ECM fiber was EC-type-dependent. The differences in fiber characteristics seen between HMEC with fibroblast and HMEC alone were absent when comparing those hydrogels with HPMEC. VNF also changed surface chemistry, and surface features [56]. In our study, the generation of new proteins and degradation of existing proteins in the cast hydrogels, as well as changes in fiber structure, are attributed to the behavior of cells, and conversely, influenced cell behavior.

Our research substantiated that fibroblast-generated newly deposited ECM, at least fibronectin and fibulin-1, facilitates ECs sprouting and tube formation. Fibronectin is an ECM protein which modulates interactions between endothelial and perivascular cells in order to modulate VNF. It is essential for vascularization, evidenced by the non-viability of mice lacking fibronectin [57]. Our study confirmed that fibronectin was mainly generated by fibroblasts adjacent to ECs. Fibronectin was also observed to be closely associated with the newly deposited collagen fibrils, as seen in our merged images of fibronectin and SHG (unpublished data). Fibulin-1 is also an ECM glycoprotein [58]. The function of fibulin-1-ECM interactions is not yet completely explored, with some studies suggesting it binds to other ECM protein like fibronectin and can promote the stabilization of ECM [59]. Also, fibulin-1 regulates cell migration such as required for VNF [60]. Our skin ECM hydrogels seeded with ECs alone contained little fibulin-1. Fibroblasts were the main contributor to the secretion of fibulin-1, and the fibulin-1 was mainly located around the ECs when the hydrogels also contained fibroblasts, similar to the pattern of fibronectin deposition. We acknowledge a limitation in our study, as we did not undertake additional experiments to delve into the potential mechanisms underlying the role of fibulin-1. In vivo, ECs attract mural, mesenchymal, cells through secretion of platelet-derived growth factor – BB (PDGF-BB). Our results do not solve the ‘chicken or egg’ question i.e. whether the fibroblast-derived fibulin-1 and fibronectin attracted ECs or that ECs attracted fibroblasts or even perhaps both. The fact that only a proportion of the fibroblasts had integrated in the vascular tubes suggests that ECs migrated to fibroblasts which subsequently engaged in intimate contact after PDGF stimulation.

## 5 Conclusion

This study elucidates the dynamic process of matrix remodeling through the interaction between fibroblasts and ECs, resulting in changes in the architectural and chemical characteristics of the ECM. Our organ-derived ECM 3D culturing model provides valuable insights into the role of fibroblasts in vessel formation and wound healing, advancing our understanding of these processes.

## Author contributions

Meng Zhang: conceptualization, methodology, investigation, formal analysis, and writing. Fenghua Zhao, Xue Zhang, Linda A. Brouwer: investigation and methodology. Janette K. Burgess, Martin C. Harmsen: conceptualization and writing. All authors have read and agreed to the published version of the manuscript.

## Declaration of generative AI and AI-assisted technologies in the writing process

During the preparation of this work, the authors used ChatGPT-3.5 (San Francisco, USA) in order to improve language. After using this tool/service, all authors reviewed and edited the content as needed and take full responsibility for the content of the publication.

## Declaration of competing interest

The authors declare that they have no known competing financial interests or personal relationships that could have appeared to influence the work reported in this paper.

## Data Availability

Data will be made available on request.
