# Supplementary figures and images for: Fibroblasts alter the physical properties of dermal ECM-derived hydrogels to create a pro-angiogenic microenvironment
Source: Mater Today Bio. 2023 Oct 24;23:100842. doi: 10.1016/j.mtbio.2023.100842 (PMC10628774; doi:10.1016/j.mtbio.2023.100842)

A

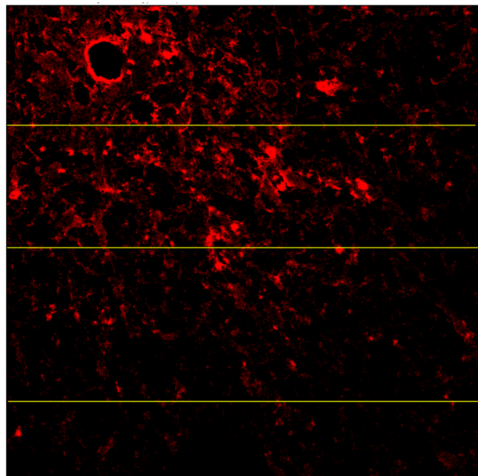

Figure S1

A

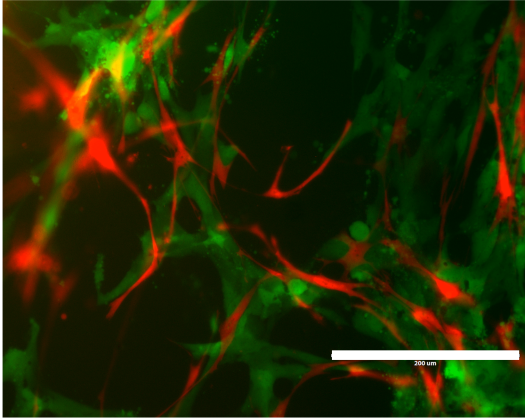

B

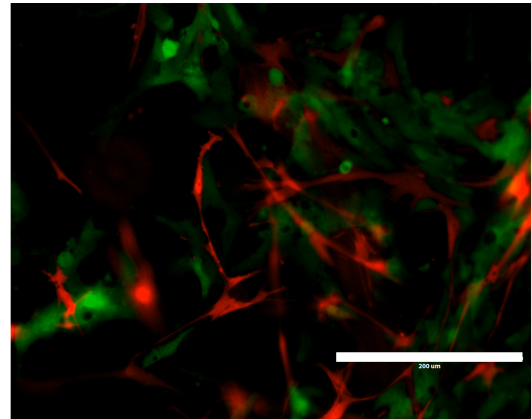

C

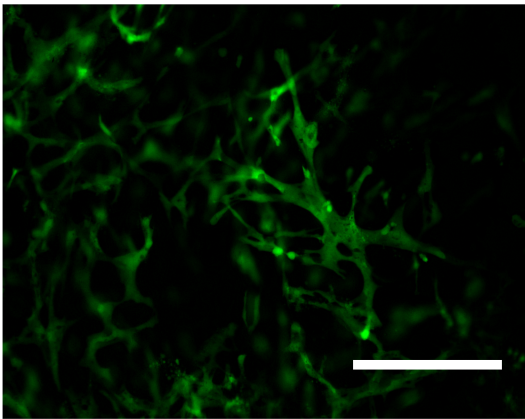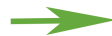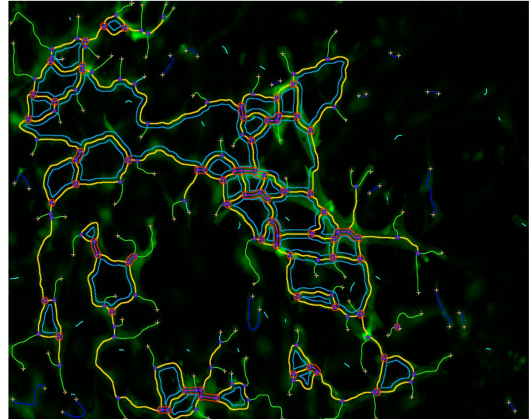

Figure S2

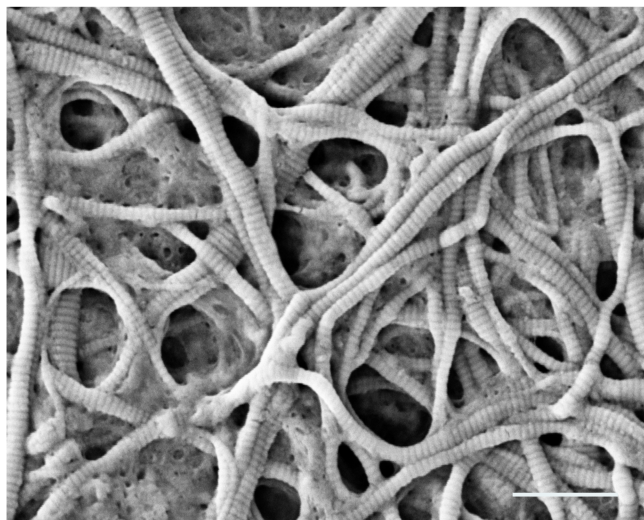

**Figure S3**

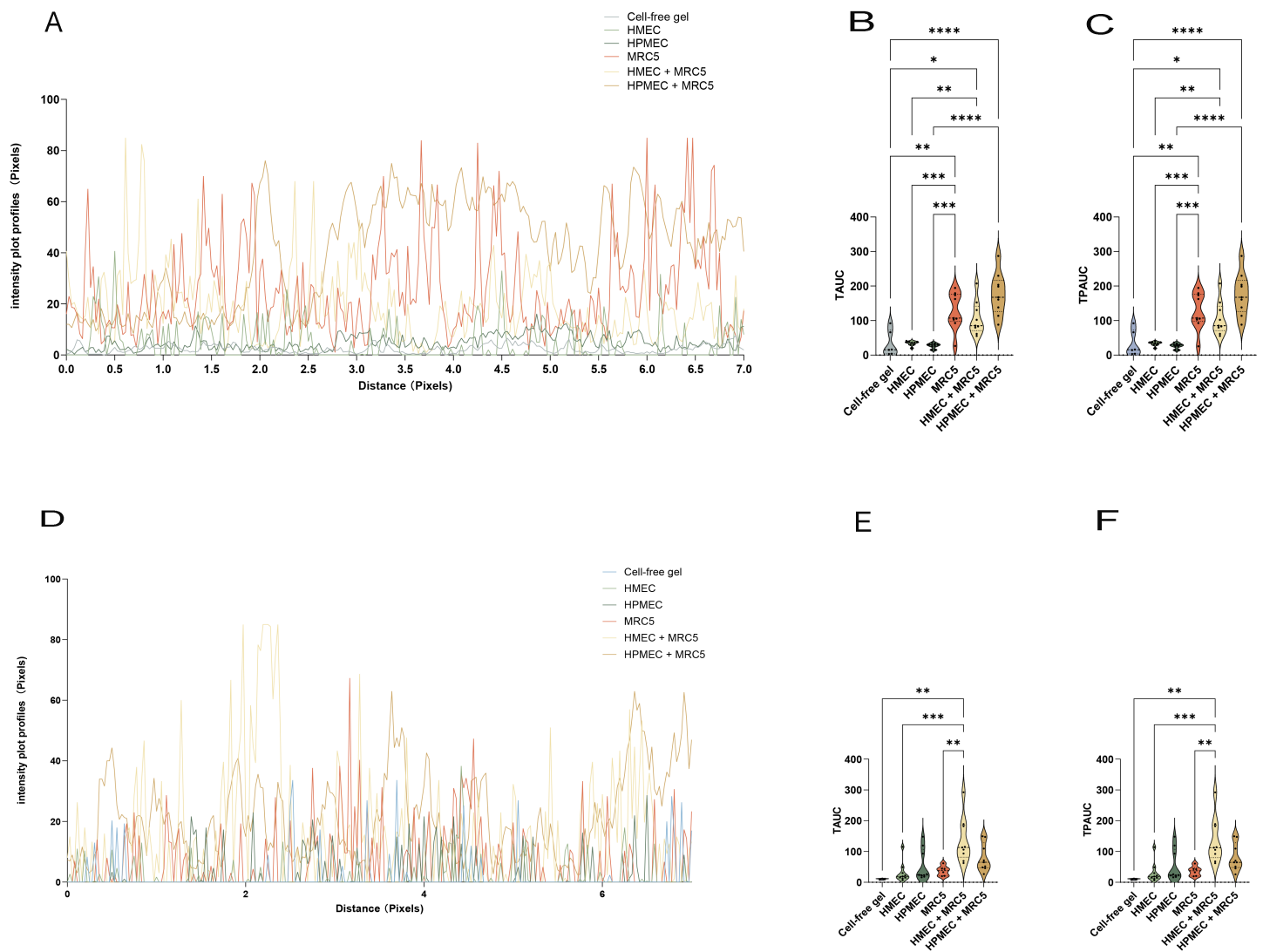

**Figure S4**

Supplement: Multimedia component 1 [file mmc1.pdf]
